# 2191. An Assessment of Penicillin Allergy Documentation among Patients undergoing Elective Surgeries and Ambulatory Procedures

**DOI:** 10.1093/ofid/ofad500.1813

**Published:** 2023-11-27

**Authors:** David Banach, Katherine Zavez, Carol Schramm, Meagan Zolla, Kate Baron, Eileen J Carter

**Affiliations:** UConn Health, Farmington, Connecticut; University of Connecticut, Storrs, Connecticut; UConn Health, Farmington, Connecticut; UConn Health, Farmington, Connecticut; University of Connecticut School of Nursing, Storrs, Connecticut; University of Connecticut, Storrs, Connecticut

## Abstract

**Background:**

A documented allergy to a penicillin class antibiotic can have a significant impact on perioperative antibiotic prescribing and is associated with an increased risk of post-operative adverse events. Comprehensive penicillin allergy histories enable risk stratification to influence future antibiotic prescribing. We evaluated the documentation of penicillin allergies among patients undergoing elective surgeries and ambulatory procedures to identify opportunities to improve allergy assessments and documentation in these settings.

**Methods:**

This was a retrospective chart review of unique records of patients who underwent elective surgeries and ambulatory procedures at UConn Health from December 2, 2022 – January 24, 2023, and had a documented allergy to a penicillin class antibiotic at the time of the surgery or procedure. Information on the documented details of the allergy, including type and timing of the reaction and who documented the allergy was recorded and analyzed.

**Results:**

Among 233 unique records reviewed, most penicillin allergies were initially documented by a medical assistant (n=135, 58%), and not subsequently updated after initial documentation (n=211, 91%). Symptoms of allergy were documented for 177 (76%) of records, with the most common symptoms being hives/rash (n=106, 46%), gastrointestinal symptoms (n=27, 12%) and anaphylaxis (n=26, 11%). Symptoms of allergy were unknown or missing for 56 (24%) of records. In 5 (3%) of records, clinicians documented when the patient’s reaction occurred (childhood) and in 2 (1%) of records, clinicians indicated the patient tolerates cephalexin or amoxicillin.

**Conclusion:**

Among patients undergoing elective surgeries and ambulatory procedures, we found medical assistants documented most penicillin allergy records. Penicillin allergy records were rarely updated and contained scant information for risk stratification. Efforts are needed to improve the documentation of penicillin allergies in outpatient pre-operative and pre-procedural settings.

**Disclosures:**

**All Authors**: No reported disclosures

